# CUT OFF VALUES FOR WAIST CIRCUMFERENCE TO PREDICT OVERWEIGHT IN BRAZILIAN ADOLESCENTS, ACCORDING TO PUBERTAL STAGING

**DOI:** 10.1590/1984-0462/;2019;37;1;00003

**Published:** 2018-07-26

**Authors:** Ivete Alves dos Santos, Maria Aparecida Zanetti Passos, Isa de Pádua Cintra, Mauro Fisberg, Roberta de Lucena Ferreti, Aline De Piano Ganen

**Affiliations:** a Centro Universitário São Camilo, São Paulo, SP, Brasil.; b Universidade Federal de São Paulo, São Paulo, SP, Brasil.

**Keywords:** Waist circumference, Adolescent, Puberty, Overweight, Circunferência da cintura, Adolescente, Puberdade, Sobrepeso

## Abstract

**Objective:**

To establish waist circumference cut off points according to pubertal staging to identify overweight in adolescents.

**Methods:**

Longitudinal study approved by the Ethics Research Committee and conducted with 557 adolescents, aged 10 to 15 years old, selected from public schools. Waist, arm, neck and hip circumferences, body fat percentage, body mass index (BMI), height and blood pressure were measured. Pubertal staging was evaluated by Tanner self assessment scale. The Receiver Operating Characteristic Curve (ROC curve) was used to determine predictive power, sensitivity, specificity and waist circumference cut off points to detect overweight.

**Results:**

There was a positive correlation between waist circumference and weight, BMI, upper arm and hip circumferences, waist-to-hip ratio and blood pressure in both sexes. Cut off points for waist circumference according to pubertal stage as related to overweight in adolescents with the best performances in ROC curve were: 71.65 cm for prepubescent girls, 67.90 cm for pubescent girls, 70.25 cm for post pubescent girls, and 66.45 cm for pubescent boys. Age, weight, height, BMI, body fat percentage, arm and hip circumferences were associated to altered waist circumference.

**Conclusions:**

The establishment of cut off points for waist circumference according to pubertal staging was proven a good means to identify overweight. These cut off points can be considered reliable for the Brazilian adolescent population, as the isolated use of chronological age in adolescents may underestimate their nutritional status.

## RESUMO

**Objetivo:**

Elaborar pontos de corte de circunferência da cintura de acordo com o estadiamento puberal para identificar sobrepeso em adolescentes.

**Métodos:**

Trata-se de um estudo longitudinal, aprovado pelo Comitê de Ética em Pesquisa, com 557 adolescentes, de 10 a 15 anos de idade, selecionados em escolas públicas. Aferiram-se as medidas de circunferência de cintura, braço, pescoço e quadril, percentual de gordura, massa corporal, estatura e pressão arterial. Para determinar a maturação sexual, foi utilizada a autoavaliação da escala de Tanner. Aplicou-se a curva ROC (Receiver Operating Characteristic Curve) para determinar: poder preditivo, sensibilidade, especificidade e os pontos de corte de circunferência de cintura para identificação de sobrepeso.

**Resultados:**

Observou-se correlação positiva entre circunferência de cintura e massa corporal, índice de massa corporal (IMC), circunferência do braço e quadril, razão cintura/quadril e pressão arterial em ambos os sexos. Os pontos de corte para circunferência de cintura de acordo com o estadiamento puberal para identificação de sobrepeso que apresentaram maior desempenho na curva ROC foram: 71,65 cm para meninas pré-púberes, 67,90 cm para meninas púberes, 70,25 cm para meninas pós-púberes e 66,45 cm para meninos púberes. Faixa etária, massa corporal, estatura, IMC, porcentagem de gordura, circunferência do braço e do quadril foram considerados fatores preditores da circunferência da cintura alterada.

**Conclusões:**

Os pontos de corte de circunferência da cintura de acordo com o estadiamento puberal demonstraram excelente desempenho para a identificação de sobrepeso, podendo ser considerados fidedignos para a população de adolescentes brasileiros, uma vez que utilizar apenas a idade cronológica na adolescência pode subestimar o estado nutricional.

## INTRODUCTION

According to the World Health Organization (WHO),^1^ adolescents are individuals aged between 10 years and 19 years, 11 months and 29 days, characterized as going through intense physiological, psychosocial, behavioral, cultural, and emotional changes.^2^ ^,^ ^3^ In puberty, hormonal changes responsible for the appearance of secondary elements and physical changes take place. Therefore, the degree of physical development of adolescents cannot be solely determined by chronological age, since it is influenced by other environmental and intrinsic factors.^4^

Adolescence can be considered a critical time for the onset of obesity. Studies have shown that adolescents have inadequate food preferences, with high consumption of processed and ultra-processed foods, which may favor diseases such as obesity, diabetes, hypertension, and metabolic syndrome.^5^ ^,^ ^6^ ^,^ ^7^ Obesity is explained as an inflammatory disease of multifactorial etiology, resulting from imbalance of energy balance and promoting accumulation of adipose tissue. Fat accumulation in the abdominal region implies increase in inflammatory adipokines, which, in turn, intensifies the risk of insulin resistance and the frequency of cardiovascular diseases.^5^

Thus, evaluating anthropometric measures associated with cardiometabolic markers becomes essential to identify risks of developing non-communicable chronic diseases. Waist circumference has been considered an important data for this assessment, whose measurement is low-cost and has excellent association with body image exams and cardiovascular risk markers.^5^ ^,^ ^6^ ^,^ ^7^ Increase in children and adolescents’ waist circumference, with cut-off points suggested by three authors in different years, showed a good correlation with dyslipidemia, hypertension, insulin resistance, and metabolic syndrome.^7^ ^,^ ^8^ ^,^ ^9^ A longitudinal study reported an increase in body fat percentage of post-pubertal girls, with strong correlation between the weight gain and waist circumference at the age of menarche.^10^

Thus, nutritional status assessment should be performed in adolescents according to their pubertal staging, since changes in it strongly influence the process of sexual maturation. Excessive visceral adiposity has been associated with early menarche^10^ and negative impacts on bone mass and bone mineral density in both genders. Higher growth rate and advanced bone aging have also been identified, including the progression of pubertal events in overweight adolescents, although it has not resulted in benefits to final stature gain.^2^ ^,^ ^11^ ^,^ ^12^

In addition, it is vital to monitor nutritional status and to recognize factors leading to such changes from a public-health standpoint, which requires interventions to reduce risks and improve the quality of life of children and adolescents.^13^

The lack of studies associating anthropometric markers and indicators of cardiometabolic risks with pubertal development is evidenced. That being said, the main purpose of this study was to establish cut-off points for waist circumference indicating overweight in Brazilian adolescents according to pubertal staging.

## METHOD

This was a longitudinal observational study conducted for three years, abiding by the guidelines for research involving human beings, contemplated by the National Health Council Resolution.^13^ The project was also approved by the Research Ethics Committee of *Centro Universitário São Camilo*, protocol number 60/2016. To that end, the informed consent form was obtained from the directors of the educational units, from parents or guardians of participants, and from adolescents taking part in it.

After obtaining the list of schools under the jurisdiction of the General Secretariat of Education of the State of São Paulo, according to the 2010 School Census, a draw was made to find a sample representative of the city of São Paulo. After the management’s approval, a meeting was held with teachers to orient them on how to approach adolescents and collect data. Afterwards, the free and informed consent form was collected from parents and guardians, and the agreement form was signed by the adolescents. A total of 557 adolescents aged 10 to 15 years, from three public schools in the city of São Paulo, were selected for meeting the following inclusion criteria: age between 10 and 15 years, enrolled in the last year of primary education to the third year of high school. Exclusion criteria were: be taking part in a weight loss program, presenting chronic or mental illness, using medications that could interfere with body composition and blood pressure (BP), as well as gestation and lactation.

All the professionals involved in the study were trained and qualified to make anthropometric measurements, with evaluation of repeatability of measurements at three moments of the study: in the first semester of each study year, in separate forms, in order to prevent the evaluator from accessing the values previously recorded and thus avoid memory bias. Variables collected were: body weight (kg); height (cm), to calculate body mass index (BMI); arm, hip, neck, and waist circumferences (AC, HC, NC, WC) (cm); and BP.

Weight was measured in a portable digital scale of the brand Seca^®^ (Seca Brasil, Cotia, São Paulo, Brazil), with a capacity for 150 kg. The adolescents were weighed while standing on the platform, barefoot, in light clothing and in a firm position with their arms along the body, with one decimal place being considered.^14^

Height was obtained in a digital wall stadiometer of the brand Seca^®^ (Seca Brasil, Cotia, São Paulo, Brazil), at 90º to the floor, fixed to a wall without skirting board. The participant was instructed to stand barefoot, without a cap and with loosen hair, eyes and ears lined horizontally, inhaling, and with their back to the instrument.

BMI was then calculated using weight and height values.^14^ For this purpose, the weight in kilograms (kg) was divided by the square height in meters (m). Standards established by WHO in 2007^15^ were used, considering the following BMI Z scores for age: <-3: accentuated thinness; ≥-3 and <-2: thinness; ≥-2 and ≤+1: eutrophy; ≥+1 and ≤+2: overweight; ≥+2 and ≤+3: obesity; >+3: severe obesity.

Arm circumference (AC) was measured with an inelastic tape (Seca Brasil, Cotia, São Paulo, Brazil), with the teenager standing up with arms positioned along the trunk, palms facing their thighs, wearing sleeveless clothes to allow full exposure of the shoulder area. To locate the midpoint, the left elbow was flexed at 90º, the distance between the acromion and the olecranon being measured and the midpoint between both extremes marked. The measuring tape was positioned perpendicular to the long axis of the arm at the midpoint marked, and the circumference was measured from the nearest to 0.1 cm.^16^

For the hip circumference (HQ), in cm, the same measuring tape was used to check the perimeter of the hip in the area with the largest apparent circumference of the gluteal region.^17^ Waist-to-hip ratio (WHR) was calculated by dividing the waist circumference (WC) (cm) by the perimeter of the hip (cm).^18^

Tricipital and subscapular skinfolds thickness was also checked for body fat percentage (BF%) using the equation proposed by Slaughter et al.^19^ and classified by Lohman.^20^ WC (in cm) was obtained with the same measuring tape (Seca Brasil, Cotia, São Paulo, Brazil), from the midpoint between the last costal arch and the iliac crest.^17^

Finally, for neck circumference (NC, in cm), participants would stand, erect and with their head positioned in the Frankfurt horizontal plane. The upper end of the measuring tape was placed just below the laryngeal prominence and positioned perpendicular to the long axis of the neck at the thyroid cartilage level, and the circumference was measured from the nearest to 0.1 cm.^21^

BP was measured by an indirect method, with an auscultatory technique and a mercury or aneroid sphygmomanometer, both calibrated. Patients’ preparation followed a protocol, in compliance with the 5th Brazilian Guidelines for Hypertension, 2006.^22^ Three measures were taken, with a 1-minute interval between each of them, and the average value of the last two was considered the adolescent’s BP. BP values were classified as suggested by the International Diabetes Federation (IDF),^23^ according to which arterial hypertension is identified by systolic blood pressure values ≥130 mmHg and diastolic blood pressure ≥85 mmHg for individuals aged 10 to 16 years or older.

By the self-evaluation technique, pubertal staging was defined based on the classification proposed by Tanner,^3^ which uses five levels to classify breast development (M1, M2, M3, M4, M5) for girls and the development of genitalia (G1, G2 , G3, G4, G5) for boys, being considered prepubertal adolescents those who report being in M1 and G1, pubertal from M2 to M4 or G2 to G4, and post-pubertal M5 and G5. At this session, after each student had their anthropometric data measured, they were taken to an isolated place of the room where the researcher explained the importance of evaluating sexual maturation and presented them boards with images of breasts/genitalia and pubic hair. This procedure was taken very carefully and strictly so as not to cause the student to be embarrassed or uncomfortable and end up pinpointing any stage indistinctly. For data analysis, we decided to use development of organs for both gender, since pubic hairiness on its own can be influenced by ethnic characteristics.

Upon statistical analysis, the results were described in absolute numbers and proportions for categorical data, and in mean and standard deviation (SD) for numerical data. In order to verify data distribution, the Kolmogorov Smirnov test was applied. Comparison of numerical variables between genders was performed with the Mann-Whitney test, and the Kruskal-Wallis test was applied to compare three or more groups. The McNemar-Bowker, Friedman and Wilcoxon tests were used to compare the variables obtained in the three evaluation sessions. For comparing the longitudinal measures from the three evaluations carried out in each year between groups, the analysis of variance for repeated measures (ANOVA) was used, followed by Tukey’s *post-hoc* test.

The correlation between waist circumference and independent variables was determined by the Pearson’s correlation test. The predictive power and waist circumference cut-off points for overweight prevention were established using the Receiver Operating Characteristic Curve (ROC curve). The total area under the ROC curve between waist circumference and overweight/obesity prevention was identified. The greater the area under the ROC curve, the greater the discriminatory power and the 95% confidence interval (95%CI). Sensitivity and specificity of waist circumference cut-off points were also calculated.

To identify the association between WC and independent variables, a univariate logistic regression analysis was performed. Binary regression was used to estimate odds ratio (OR) for the risk of altered waist circumference as a result.

All calculations were performed in the Statistical Package for Social Sciences (SPSS) version 20.0 (IBM, New York, New York, USA), and the significance level was set at p<0.05.

## RESULTS

During the study period, BMI, BF%, HC and NC were statistically different in both genders. Mean BMI, HC, and BF% values were higher in girls, and NC was higher in boys. In the last year of study, BF% was perceived higher in girls, while the mean progressive increase in height was higher in boys over the three years. Across three years of research, pubertal staging progressed for subjects of both genders. In the first year, most of participants were in the pubertal stage, while in the last year they were found to be in post-pubertal phase, with only one girl in the pre-pubertal phase (Table 1).

**Table 1:** Descriptive analysis of anthropometric variables (mean and standard deviation) of adolescents according to gender, during the study.

		First year				Second year				Third year			
		Total	Boys	Girls	p-value^a^	Total	Boys	Girls	p-value^a^	Total	Boys	Girls	p-value^a^
Age (years)	$\overset{-}{\chi }$	12.00	12.10	11.90	0.371	13.00	13.10	13.00	0.497	13.80	13.90	13.80	0.636
	SD	1.10	1.20	1.10		1.10	1.20	1.10		1.10	1.20	1.10	
Weight (kg)	$\overset{-}{\chi }$	46.60	45.80	47.30	0.176	51.50	51.00	51.80	0.497	54.50	54.30	54.60	0.835
	SD	12.60	13.50	11.90		13.40	14.70	12.30		12.90	14.10	11.90	
Height (cm)	$\overset{-}{\chi }$	152.10	151.80	152.20	0.615	156.80	157.50	156.10	0.072	160.10	161.40	159.00	0.001
	SD	9.30	10.70	8.00		8.60	10.40	6.50		8.20	9.90	6.10	
BMI (kg/m^2^)	$\overset{-}{\chi }$	19.90	19.50	20.30	0.039	20.80	20.40	21.10	0.065	21.20	20.70	21.60	0.023
	SD	4.20	4.00	4.40		4.60	4.80	4.50		4.30	4.20	4.40	
BF%	$\overset{-}{\chi }$	15.60	9.80	20.60	<0.001	18.90	13.60	23.20	<0.001	20.00	14.10	24.90	<0.001
	SD	11.60	10.70	10.10		10.40	11.20	7.50		14.70	10.40	16.00	
AC (cm)	$\overset{-}{\chi }$	23.30	23.10	23.40	0.272	24.20	23.90	24.40	0.157	25.10	24.70	25.50	0.041
	SD	4.10	4.00	4.10		4.60	4.50	4.60		4.50	4.10	4.70	
WC (cm)	$\overset{-}{\chi }$	68.40	68.30	68.40	0.935	68.20	68.20	68.20	0.946	70.50	70.60	70.40	0.863
	SD	11.70	11.80	11.50		10.90	11.20	10.70		10.70	11.30	10.10	
HC (cm)	$\overset{-}{\chi }$	82.90	81.10	84.40	0.001	85.50	83.80	86.90	0.002	88.50	86.40	90.20	<0.001
	SD	12.00	10.30	13.20		12.00	11.50	12.30		11.00	11.30	10.40	
NC (cm)	$\overset{-}{\chi }$	30.00	30.80	29.40	<0.001	30.60	31.40	30.00	<0.001	31.10	31.80	30.50	<0.001
	SD	4.40	4.40	4.30		3.60	4.70	2.20		2.80	3.10	2.40	
WHtR	$\overset{-}{\chi }$	0.55	0.56	0.55	0.406	0.43	0.43	0.43	0.469	0.44	0.43	0.44	0.289
	SD	0.03	0.03	0.02		0.06	0.06	0.06		0.06	0.06	0.06	
WHR	$\overset{-}{\chi }$	1.03	1.06	1.01	0.002	0.80	0.81	0.79	0.348	0.79	0.81	0.78	<0.001
	SD	0.16	0.13	0.17		0.20	0.06	0.27		0.07	0.05	0.07	
Tanner: prepubertal	n	22.00	-	22.00		5.00	-	5.00		1.00	-	1.00	
	%	3.90	-	7.30		0.90	-	1.70		0.20	-	0.30	
Tanner: pubertal	n	505.00	256.00	249.00	0.001*	495.00	256.00	239.00	0.001*	474.00	256.00	218.00	0.001*
	%	90.70	100.00	82.70		88.90	100.00	79.40		85.10	100.00	72.40	
Tanner: post-pubertal	n	30.00	-	30.00		57.00	-	57.00		82.00	-	82.00	
	%	5.40	-	10.00		10.20	-	18.90		14.70	-	27.20	

^a^ *t*-test for independent samples: *p<0.05; SD: standard deviation; BMI: body mass index; BF%: body fat percentage; AC: arm circumference; WC: waist circumference; HC: hip circumference; NC: neck circumference; WHtR: waist-to-height ratio; WHR: waist-to-hip ratio.

A significant correlation was found between WC and the other variables, except for WHR in girls. A strong positive correlation between WC and body mass, BMI, HC, AC, NC and waist-to-height ratio (WHtR) in both sexes (Table 2).

**Table 2:** Correlation between waist circumference, anthropometric variables and blood pressure levels in adolescents, according to gender.

	Total		Boys		Girls	
	r^a^	p-value	r^a^	p-value	r^a^	p-value
Age (years)	0.20	<0.001	0.17	0.006	0.23	<0.001
Weight (kg)	0.84	<0.001	0.86	<0.001	0.83	<0.001
Height (cm)	0.35	<0.001	0.39	<0.001	0.28	<0.001
BMI (kg/m^2^)	0.85	<0.001	0.88	<0.001	0.84	<0.001
Body fat percentage	0.51	<0.001	0.58	<0.001	0.61	<0.001
Neck circumference (cm)	0.99	<0.001	0.99	<0.001	0.99	<0.001
Hip circumference (cm)	0.82	<0.001	0.87	<0.001	0.79	<0.001
Arm circumference (cm)	0.79	<0.001	0.83	<0.001	0.76	<0.001
WHtR	0.74	<0.001	0.71	0.001	0.78	<0.001
WHR	-0.09	0.031	-0.26	<0.001	-0.02	0.195
Systolic blood pressure (mmHg)	0.26	<0.001	0.27	<0.001	0.26	<0.001
Diastolic blood pressure (mmHg)	0.26	<0.001	0.21	0.001	0.33	<0.001

^a^Pearson’s correlation, performed according to variables’ mean values for all three years of study; BMI: body mass index: WHtR: waist-to-height ratio; WHR: waist-to-hip ratio.

When analyzing the cut-off points for WC in girls, there were not enough individuals in the prepubertal stage in the second and third years of study to build the ROC curve. The cut-off points for WC for girls with better performance, that is, area under the ROC curve with high sensitivity and specificity to identify overweight, were 71.65 cm for prepubertal subjects, 67.9 cm for pubertal subjects, and 70.25 cm for post-pubertal subjects. For pubertal boys, the cut-off was 66.45 cm (Table 3).

**Table 3:** Results of waist circumference cut-off points in identifying overweight/obesity, according to pubertal staging and gender, over the three years of study.

	Area under the ROC curve	95%CI	Cut-off point	S	E
First year					
Boys					
Prepubertal	-	-	-	-	-
Pubertal	0.91	0.87-0.94	66.65	0.89	0.74
Post-pubertal	-	-	-	-	-
Girls					
Prepubertal	0.98	0.95-1.00	71.65	1	0.95
Pubertal	0.87	0.82-0.92	69.15	0.82	0.83
Post-pubertal	0.91	0.80-1.00	70.50	0.72	0.88
Second year					
Boys					
Prepubertal	-	-	-	-	-
Pubertal	0.9	0.85-0.94	68.75	0.82	0.86
Post-pubertal	-	-	-	-	-
Girls					
Prepubertal	-	-	-	-	-
Pubertal	0.89	0.85-0.93	67.90	0.81	0.82
Post-pubertal	0.91	0.84-0.98	68.25	0.78	0.83
Third year					
Boys					
Prepubertal	-	-	-	-	-
Pubertal	0.91	0.87-0.94	69.90	0.82	0.83
Post-pubertal	-	-	-	-	-
Girls					
Prepubertal	-	-	-	-	-
Pubertal	0.84	0.78-0.90	70.05	0.71	0.72
Post-pubertal	0.92	0.86-0.97	70.25	0.82	0.84

ROC curve: Receiver Operating Characteristic Curve; 95%CI: 95% confidence interval; S: sensitivity; E: specificity.

In Table 4, the adjusted logistic regression analysis shows that age, body mass, stature, BMI, BF%, AC and HC were considered predictors of altered WC. The association between altered WS and BMI (OR=2.29, 95%CI 1.99**-**2.62; p≤0.001), NC (OR=3.09, 95%CI 1.65**-**2.82; p≤0.001), and AC (OR=2.11, 95%CI; 1.87**-**2.39, p≤0.001).

**Table 4:** Association between waist circumference cut-off point for identification of overweight and independent variables by logistic regression model.

	Waist circumference*	
	OR (95%CI)	p-value
Gender		0.570
Female	1.00	
Male	0.90 (0.65-1.26)	
Age (years)	1.34 (1.15-1.56)	<0.001
Weight (kg)	1.27 (1.22-1.32)	<0.001
Height (cm)	1.08 (1.06-1.11)	<0.001
BMI (kg/m^2^)	2.29 (1.99-2.62)	<0.001
Body fat percentage	1.13 (1.10-1.16)	<0.001
Neck circumference (cm)	3.09 (1.65-2.82)	<0.001
Hip circumference (cm)	2.11 (1.87-2.39)	<0.001
Arm circumference (cm)	1.29 (1.23-1.34)	<0.001
Waist-to-hip ratio	0.05 (0.05-0.59)	0.017
Systolic blood pressure (mmHg)^#^		
Normal	-	
Hypertension 1	-	
Diastolic blood pressure (mmHg)		0.231
Normal	1	
Hypertension 1	1.21 (0.87-1.98)	

*Adjusted for year of study; OR: *odds ratio*; 95%CI: 95% confidence interval; BMI: body mass index; ^#^insufficient number of subjects with hypertension, with no possibility of logistic regression for this variable.

## DISCUSSION

The most important finding of our research is the establishment of cut-off points to identify overweight according to pubertal staging, since chronological age may not represent a safe parameter for nutritional evaluation and characterization of the nutritional status of adolescents at this stage of development. Associating pubertal staging with the cut-off point of a simple anthropometric measure to identify the risk of overweight allows the health professional a better understanding and more assertive clinical management.^24^

As pubertal staging progresses, one notes the appearance of typical characteristics in body composition, according to gender. Upon temporal evaluation, the highest BF% was found in girls in the last year of study. This difference in body composition, in addition to higher BMI and HC, can be considered a result of pubertal events, with greater acquisition of muscle mass in males and greater fat deposition in the hip region and BF% in females, which give them gender-like forms. In this period of adolescence, nutritional status may strongly influence pubertal development, with emphasis to body fat deposition and distribution.^25^

An epidemiological study reported the role of adipose tissue in triggering and maintaining reproductive activity.^25^ Adrenal and ovarian androgen production are elevated when there is obesity. The aromatization of androgens, which converts them into estrogens, occurs in adipose tissue for both gender and is strongly related to body weight.^26^ A recent study on the impact of excess body fat on bone remodeling of adolescents showed that body fat promotes a negative impact on bone mass, as well as bone aging and pubertal events’ acceleration in both genders.^11^

Research has shown that abdominal obesity, even without other risk factors associated, is strongly related to the development of metabolic syndrome in young populations. Thus, WC can be considered an efficient indicator of abdominal obesity and, consequently, of cardiovascular risk among children and adolescents.^6^ ^,^ ^27^ ^,^ ^28^ In a recent study with obese adolescents, a positive correlation between WC and BMI was reported in both genders, which corroborates our results.^6^

Supporting our findings, Mazicioglu et al.^29^ also identified a strong correlation between WC and BMI, concluding that its cut-off points can be used to identify risk of overweight in children. Another study reported females with significantly higher WHR, suggesting association with metabolic alterations related to glucose and arterial hypertension, and confirming its importance in diagnosing chronic diseases. In the same study, the mean WC in males was higher and there was a relation between very high WC and increased NC.^21^ Hence, WC alterations reflected the pattern of fat distribution and changes in risk factors for cardiovascular diseases in males. Some authors have identified a strong association between WC and cardiovascular events.^29^ ^,^ ^30^

WC has been used as a reliable instrument to evaluate central obesity in clinical practice, because it is simple to measure, is low-cost, and has excellent correlation with abdominal imaging tests and association with cardiovascular disease risk.^30^ Important to note that computed tomography (CT) scan and magnetic resonance imaging (MRI) are considered gold standard to assess body fat distribution. Dual X-ray absorptiometry (DEXA), in turn, measures total body fat with high accuracy and low radiation, but does not distinguish intra-abdominal from subcutaneous fat.^30^

IDF^23^ proposes WC as a diagnostic data for adolescents, since it has been referred to as an important predictor of metabolic syndrome and cardiovascular disease risk.^26^ ^,^ ^27^ However, WC cut-off points adopted by this guideline for individuals older than 16 years are >90 cm for male adolescents and >80 cm for female adolescents. These cutoff values were based on a South Asian population and, for children under 16 years of age, cut-off points are age-specific and gender-specific only.^23^

The cut-off points found over the three years of our study for post-pubertal girls were lower than those adopted by IDF. Thus, it can be inferred that cut-off points not taking into account pubertal staging and ethnicity may underestimate a possible nutritional and cardiometabolic risk. It is therefore believed that the cut-off points identified by the present research can be more suitable to our population, since they were obtained based on pubertal development staging and established on a sample of Brazilian individuals.

An international study conducted with children and adolescents reported WC reference values similar to those obtained in our study.^8^ In contrast, two other investigations had cut-off points subtly lower for girls compared to our findings for this gender.^31^ ^,^ ^32^ National studies conducted with adolescents have also reported WC cut-off points similar to ours.^10^ ^,^ ^33^ Thus, together, these results suggest WC to be an important anthropometric indicator of overweight, but this measure should be evaluated as linked to the pubertal development phases of subjects, and also considering the ethnicity of the studied population.

Some limitations should be taken into account when interpreting results, including the larger number of girls compared to boys in the sample. When categorizing individuals according to pubertal staging, we noticed an insufficient number of individuals in the prepubertal stage, making it impossible to build the WC ROC curves.

However, it should be noted that the longitudinal design of this study made it possible to follow the evolution of anthropometric data and pubertal staging changes, resulting in the establishment of cut-off points based on pubertal development, which promotes major changes in body composition during adolescence, and thus allowing a more accurate and reliable tool to identify overweight in the Brazilian adolescent population.

Conclusion is that WC cut-off points applied to anthropometric evaluation of adolescents are an excellent screening tool for early identification of overweight and obesity, helping to target food reeducation and increased physical activity programs as means of preventing comorbidities.
